# Do Changes in Current Flow as a Result of Arrays of Tidal Turbines Have an Effect on Benthic Communities?

**DOI:** 10.1371/journal.pone.0161279

**Published:** 2016-08-25

**Authors:** Louise Kregting, Bjoern Elsaesser, Robert Kennedy, David Smyth, Jack O’Carroll, Graham Savidge

**Affiliations:** 1 School of Natural and Built Environment, Queen’s University Belfast, Belfast, Northern Ireland, BT7 1NN, United Kingdom; 2 Queen’s University Marine Laboratory, Queen’s University Belfast, (The Strand) Portaferry, Northern Ireland, BT22 1PF, United Kingdom; 3 School of Natural Sciences, National University of Ireland Galway, Galway, Ireland; Centro de Investigacion Cientifica y de Educacion Superior de Ensenada Division de Fisica Aplicada, MEXICO

## Abstract

Arrays of tidal energy converters have the potential to provide clean renewable energy for future generations. Benthic communities may, however, be affected by changes in current speeds resulting from arrays of tidal converters located in areas characterised by strong currents. Current speed, together with bottom type and depth, strongly influence benthic community distributions; however the interaction of these factors in controlling benthic dynamics in high energy environments is poorly understood. The Strangford Lough Narrows, the location of SeaGen, the world’s first single full-scale, grid-compliant tidal energy extractor, is characterised by spatially heterogenous high current flows. A hydrodynamic model was used to select a range of benthic community study sites that had median flow velocities between 1.5–2.4 m/s in a depth range of 25–30 m. 25 sites were sampled for macrobenthic community structure using drop down video survey to test the sensitivity of the distribution of benthic communities to changes in the flow field. A diverse range of species were recorded which were consistent with those for high current flow environments and corresponding to very tide-swept faunal communities in the EUNIS classification. However, over the velocity range investigated, no changes in benthic communities were observed. This suggested that the high physical disturbance associated with the high current flows in the Strangford Narrows reflected the opportunistic nature of the benthic species present with individuals being continuously and randomly affected by turbulent forces and physical damage. It is concluded that during operation, the removal of energy by marine tidal energy arrays in the far-field is unlikely to have a significant effect on benthic communities in high flow environments. The results are of major significance to developers and regulators in the tidal energy industry when considering the environmental impacts for site licences.

## Introduction

Renewable energy from wave and tidal technology has the potential to contribute significantly to energy security for future generations. Realistic estimates of the commercial potential of the two forms of marine energy indicate there is the possibility of up to 337GW being installed worldwide by 2050 [1]. Although the commercial development of the technology is still in the early stages, it appears that the potential exploitation of tidal energy is significantly closer to realisation than that of wave energy [2]. This reflects to some extent the higher predictability of the tidal resources on a daily basis compared to wave energy and other renewable energy resources such as solar and wind power.

Common to both tidal and wave energy extraction systems is concern regarding the potential environmental consequences of the deployment of the technology. The environmental concerns reflect differences in both the form of interaction of the system technologies with the environment and in the overall dynamics of the areas suitable for exploitation (e.g. [3, 4]). In the case of tidal energy converters (TEC), the majority either planned or constructed are characterised by large underwater rotors together with substantial supporting structures or mooring systems. Large arrays of these structures have the potential to induce significant changes in the hydrodynamic field [5]. It has been suggested that the altered hydrodynamics can have both near- (immediately around the structure) and far-field (wake and basin scale) effects which include a reduction in mean flow, tidal flow diversion, increased bottom drag and changes in tidal phasing and height [6, 7, 8]. The presence of TECs and the changed hydrodynamic field may result in direct and indirect effects on local populations of diving seabirds, fish and mammals and also, in the case of the hydrodynamics, on the benthos [3, 9, 10].

Owing to the requirement for high (>2 m/s) flow velocities, the location of TEC will be restricted to areas where seabeds are characterised by rocks, boulders, cobbles and sand [11]. Although the spatial extent of such areas is limited, the benthic communities associated with these locations are relatively pristine as they have undergone minimal exploitation or disturbance by anthropogenic influences owing to their exposed nature. To date the majority of surveys of these areas have been spatially restricted and generally undertaken for broad conservational objectives [11]. However, given present moves towards the commercial development of tidal energy, it is necessary to have a close understanding of the interplay between the physical dynamics and the biology of representative organisms in the control of benthic community structure in highly dynamic areas. Clear links have been observed between the spatial patterns for marine communities with substrate type as a direct causal result of modelled or measured current speed in which communities live (e.g. [12, 13, 14, 15]). Therefore the outcomes of investigations linking the hydrodynamics with the biology have the potential to allow predictions to be made of changes in benthic communities resulting from flow changes induced by TECs.

Preliminary investigations of the effect of a single, full-scale TEC, SeaGen, on the benthos showed somewhat surprisingly minimal influence of the device on the seabed communities [16]. Of greater importance, though, is the potential impact of arrays of TECs on the benthos. The number of turbines required to extract a significant amount of the energy is of the order of 10s to 100s. Such arrays are likely to extract much larger amounts of energy from the ambient environment resulting in potentially measurable reduction in flow velocity; however, the general lack of understanding of the interaction between arrays and the marine environment has been identified as one of the four major factors impeding the development of tidal energy as a commercial resource [17]. Advances in the application of high resolution flow models are allowing the detailed prediction of the current regime in highly dynamic areas [18] with these, in turn, allowing the design of more effective benthic surveys in the challenging conditions typifying these locations and allowing opportunity for more detailed analysis of relationships.

The aim of this study was to determine whether any observed spatial variation of macrobenthic communities could be related to changes in a natural velocity gradient in a narrow channel dominated by strong semi-diurnal tides. The approach taken was to obtain macrobenthic community data using a drop down video survey system at a number of sites in the channel spanning a range of ambient current velocities relevant to the tidal energy industry to test the null hypothesis that there was no significant difference in community structure over the gradient of flow velocities sampled. Without a tidal array in existence, this is presently the best approach in predicting the influence of TEC arrays on benthic populations located within the area of an array. This would assist regulators and the tidal site developers in site selection and environmental licensing.

## Methods

### Sampling site selection

**Fig 1:**
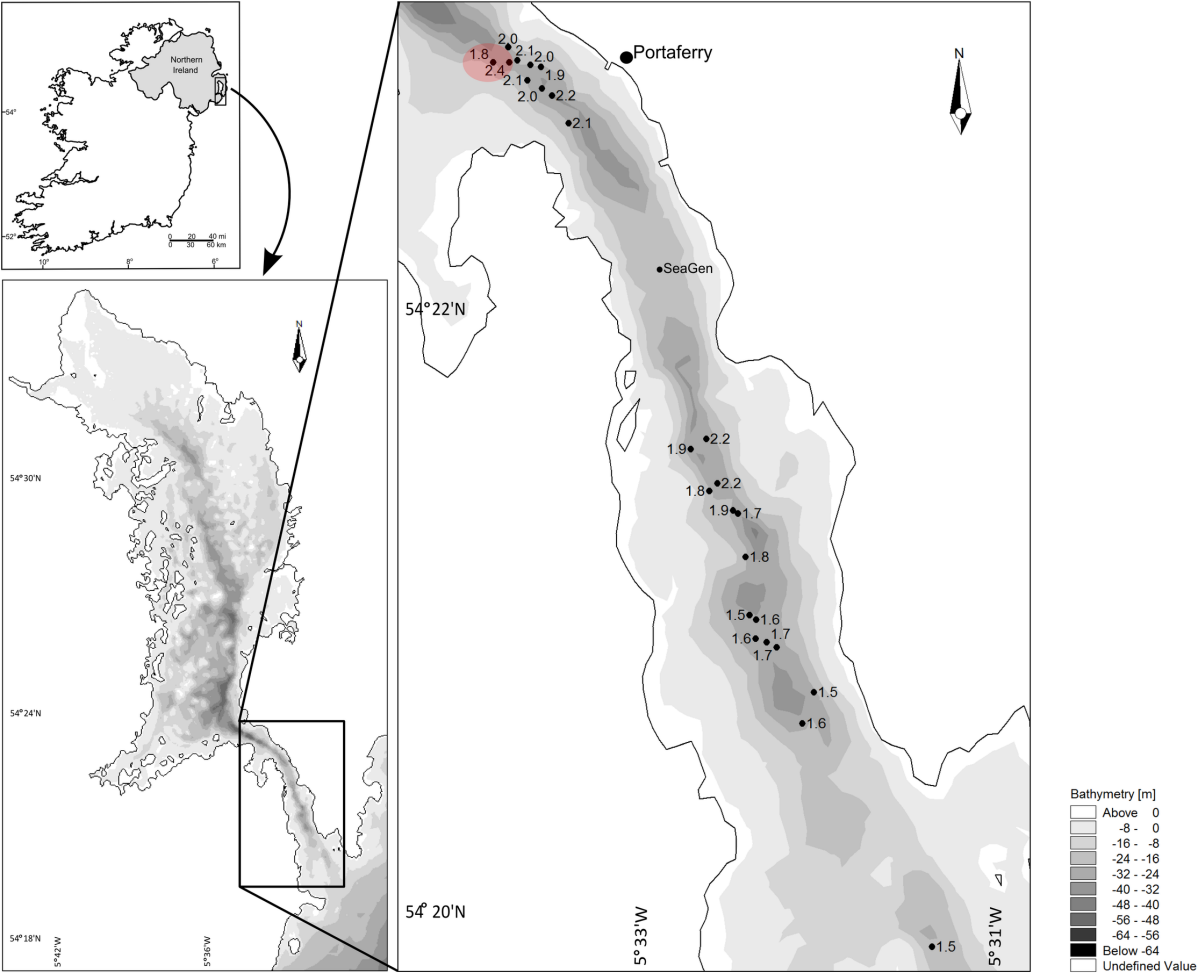
Site locations with P50 value (median velocity) within Strangford Lough Narrows. Red circle highlighting the heterogeneity of flow over a distance as little as 100 m.

Sampling was carried out in the Strangford Narrows in Northern Ireland, the location of SeaGen, the world’s first single full-scale, grid-compliant tidal energy extractor. It is representative of a site with relevant flow fields to the tidal energy industry [16] (Fig 1). The velocity range over which samples were taken was dictated by prediction of the operational flow velocities of a TEC below which the installation of a tidal turbine would be considered commercially unfavourable. To establish the range, it was first necessary to determine the velocity changes that would result from the presence of an array of TECs. It is to note here that this work was not based on the SeaGen design but rather the type of device most likely proposed for commercial development, the horizontal axis tidal turbine (HATT) [19]. Two ways to quantify this change were employed: assessment of (i) the effect of a single or array of TECs on flow velocity and (ii) the resource limit at which commercial exploitation is not viable, i.e. the point at which a commercial company would not add further turbines into a location.

When considering the potential effect of arrays of TECs on the mean flow, it is important to note that such turbines are unlikely to extract more than 50% power from the rotor swept area (the theoretical Betz Limit is 59.3%, though this is not achieved in real world applications [20]. Since hydrokinetic power is proportional to the velocity cubed, a 50% reduction in hydrokinetic power will only result in a 20% reduction in flow velocity. Further, this reduction only applies to the rotor-swept area of a device which, for present TECs, covers only a small area of the water column (the diameter of the rotor-swept area) due to technical, navigational and environmental considerations. Thus relatively large changes in power extraction by TECs will be reflected in only small changes in near-field flow speeds. Drag losses associated with the support structure of the TEC will also extract energy from the flow and assist in reducing flow velocities, although the turbulent nature of the marine environment results in fast recovery of the ambient velocity field: Savidge et al. [16] reported velocities close to the original undisturbed currents (> 95% of undisturbed velocity) at 5 rotor diameter downstream (80 m). Further it was not observed that the infrastructure of SeaGen increased velocity or turbulence close to the seabed [21]. The dynamics of wake structure and recovery in relation to TECS are poorly understood with absolute predictions likely to be device-specific. In addition, use of modelling the wake velocity as an estimator for the impact of TECs on benthic habitat is considered premature as it is not yet computationally possible to numerically model at the individual device scale to resolve blade and pile wake turbulence as well as span far-field to determine changes in hydrodynamics as a result of tidal arrays [5].

The second approach to defining a velocity range relevant to benthic interactions associated with the installation of TECs is to assess the resource limit beyond which commercial exploitation is not viable. As noted above, although tidal energy converters are still in early development, the HATT is presently the type of device most frequently proposed for commercial development [19]. HATT have characteristic cut-in speeds, i.e. velocities below which they do not function, and have design or rated speeds at which the device obtains full power output. For example the tidal turbine SeaGen at Strangford Lough has a cut-in velocity of 0.8 m/s and a rated velocity of 2.5 m/s.

Table 1 provides the percentage utilisation (power available in the flow field in relation to the power derived from the rated speed of a device) based on three hypothetical cut-in and rated velocities for three different hypothetical turbines: A, B & C. It should be noted that this is only the power available in the flow field: considerations of energy conversion efficiency to mechanical power and drive train losses are excluded here, as again they are device specific. Although no relevant data are publically available and comparable figures for the wind industry are not representative, information obtained by the authors from collaborations with various turbine developers indicate that a realistic percent utilisation for a feasible project is approximately 60% and may drop to just above 40%. This latter figure is taken as a clear minimum as it is unlikely even for a mature technology to go below this limit due to the cost of the technology, including economy of scale considerations [17]. From Table 1 it can be seen that, presently, median flow velocities of 2 m/s need to be reached or exceeded to meet the velocity requirements of current tidal energy devices. Therefore, to incorporate the predicted operating velocities of the developing current tidal turbines and associated limits, median current values ranging from 1.5 to 2.4 m/s were chosen for the study. It is to be noted that the lower limit of 1.5 m/s defines a high current environment.

**Table 1:** Resource requirements for three different hypothetical tidal energy converters (A, B & C) and percentage power availability for tidal energy generation (i.e. the power available to a turbine in real tidal flow (P50, median velocity) in relation to the hydrokinetic power obtained from the rated velocity).

		Resource of P50 (median) velocity		
	cut in / rated velocity of turbine (m/s)	1.5 m/s	2 m/s	2.4 m/s
Turbine A	0.6 / 2.2	36%	63%	74%
Turbine B	0.8 / 2.5	24%	52%	67%
Turbine C	1.2 / 2.8	17%	40%	58%

Use of the median velocity (velocity that is exceeded 50% of the time (P50)) is not commonly employed in the characterisation of high current environments; more usually peak mean neap and spring tidal current velocity values are used to define these environments. Table 2 compares three different median velocities of 1.5, 2.0 and 2.4 m/s and the approximate associated peak tidal current speeds for typical (mean) neap and spring conditions for the Strangford Narrows. From this table, it is apparent that only the neap peak flows and the median flows are similar. In contrast to peak current flow values, the median gives a measure of how often the defined flow occurs and hence is a more meaningful parameter for this study compared to the conventional peak or mean flow values.

**Table 2:** Relationship between P50 (median velocity) and typical peak neap and spring velocities for three hypothetical sites (A, B & C) in the Strangford Narrows

	Site A	Site B	Site C
P50 (median) velocity (m/s)	1.5	2.0	2.4
Mean neap tidal velocity (m/s)	1.5	2.0	2.4
Mean spring tidal velocity (m/s)	2.2	3.0	3.6

Based on the above considerations, the locations of the sites to be used for sampling of the benthos were selected from the output of the Strangford Lough current model based on MIKE 21 modelling software (DHI Water and Environment software package; www.dhisoftware.com) [18]. The hydrodynamic model has a flexible mesh and uses a cell-centred finite volume method to determine the current field by solving a depth averaged shallow water approximation (full details of the development and calibration of the model can be found in [18]). Cell size averaged approximately 50 m resulting in a total of 52,882 cells throughout the domain. The model was run over a three month period (February—April 2011) incorporating a range of neap and spring tide conditions. The outputs were recorded as Reynolds averaged velocities over 5 minute intervals; these values exclude flow fluctuation due to turbulence. The model does take into account benthic boundary layer processes even though depth averaged velocities are taken:

$$\frac{ū}{u⋆}=\frac{1}{\kappa }\;\text{ln}\left(\frac{h}{e{\text{Z}}_{0}}\right)$$ (1)

where ū is the depth averaged velocity, *u*_⋆_ is the shear velocity, κ is the von Kármán constant (0.4), *h* is the water depth, *e* is the Eulerian number and Z_0_ is the friction height. It describes the relationship between ū and *u*_⋆_ of a fully developed boundary layer. Based on a Z_0_ in the order of 0.1–0.5 m [16], the Reynolds number can be assumed to be always > 10^5^ for median velocities. This means a fully turbulent boundary layer is to be expected in the study area. While Z_0_ may vary from location to location, in general the bed in the Narrows is rough with boulders and bedrock. Thus a direct relationship between the depth averaged and the shear velocity exists, which influences the forces on the fauna and flora at the seabed.

Three replicates of each of nine defined P50 values (1.5, 1.6, 1.7, 1.8, 1.9, 2.0, 2.1, 2.2, 2.4 m/s) were randomly selected from within the Narrows except for the highest velocity, 2.4 m/s, for which only one replicate was available; no sites with P50 values of 2.3 m/s were able to be established (Fig 1). In total, 25 sites (cells) were sampled. While SeaGen has been shown to have minimal influence on seabed communities [16], cells within the 1 km radius around SeaGen were excluded from site selection in order to reduce the possibility of local bias. To control for potential effects of depth on the structure of the benthic communities, all sampling locations were restricted to a depth range of 25–30 m.

### Sampling

The 25 designated sites were sampled between 25 April and 23 May 2013 at slack water for substrate type and macrobenthic community using a drop down video survey method. A video quadrat camera system was constructed from a stainless steel frame with a GO-PRO HERO3 Black Edition (San Mateo, USA) mounted 0.4 m above the substrate to provide a quadrat size area of 0.5 × 0.5 m. Light was provided by three underwater torches (Kinetics Mini Q40 eLED Plus) placed strategically on the frame to provide an even light source across the substrate. At each site the frame was gently lowered to the substratum with the video continually recording (1080p/48 FPS with medium field of view) for approximately 5–10 seconds then lifted, moved ~ 2 m and dropped to provide 30 random quadrats within an approximate 20 m^2^ area starting at the centre coordinate of the mesh cell from which the velocities were extracted as determined from the model. Visual of the seabed during the video survey allowed for an estimate of the distance between drops. Due to tidal constraints, only one site was sampled each slack tide. Field studies in this area did not require any permit or permission and did not involve any protected or endangered species.

### Image and data analyses

The 21 best resolved still images from the 30 quadrats (630 images in total) were grabbed from the video footage using Windows Media Player. Image selection was based on clarity of the focused frame and for consistency, only one skilled marine benthic taxonomist observed the images creating a species list for the analysed frame after three separate viewings. Viewings took place with a 48 h period between sittings to avoid observer fatigue. The images were assessed for percentage cover and density of discrete individual species (identified to the nearest taxonomic level) based on a random point quadrat (100 points) methodology. Motile fauna including fish and decapods were removed from the data matrix before analysis as is typical for reef epifaunal studies [22]. The substratum composition for each quadrat was visually determined using the Joint Nature Conservation Committee (JNCC) biotope coding protocol [23].

A core assumption of the analysis is that attached sessile epifaunal distributions reflect the integration of the environmental gradients in the observational area over time. The percentage cover data and abundance were transformed to the ordinal Marine Nature Conservation Review (MNCR) SACFOR scale using the method of Connor et al. [11]. The SACFOR data were converted to a Bray-Curtis similarity matrix [24]. The null hypothesis of no significant difference in community structure was tested using a nested mixed model permutational analysis of variance (Permanova; [25, 26] in the Permanova+ package in Primer 6. The factor Site (the 20 m × 20 m area sampled on a particular tidal cycle) was random and nested within the fixed factor Velocity (the ordinal level of median velocity predicted for that sampling site in the hydrodynamic model). Species characterising the different levels of velocity were identified using the exploratory data analysis Simper [27, 28].

## Results

A spatially heterogeneous flow can be observed in the Narrows with variation in flow speeds of almost 0.5 m/s over distances as small as 50–100 m (see Fig 1). As expected, greatest flow velocities were observed in the narrowest part of the channel, near Portaferry, with velocities ranging from 1.8 to 2.4 m/s in this area. Lowest velocities were observed towards the entrance of the Narrows where the channel is at its widest (Fig 1).

**Fig 2:**
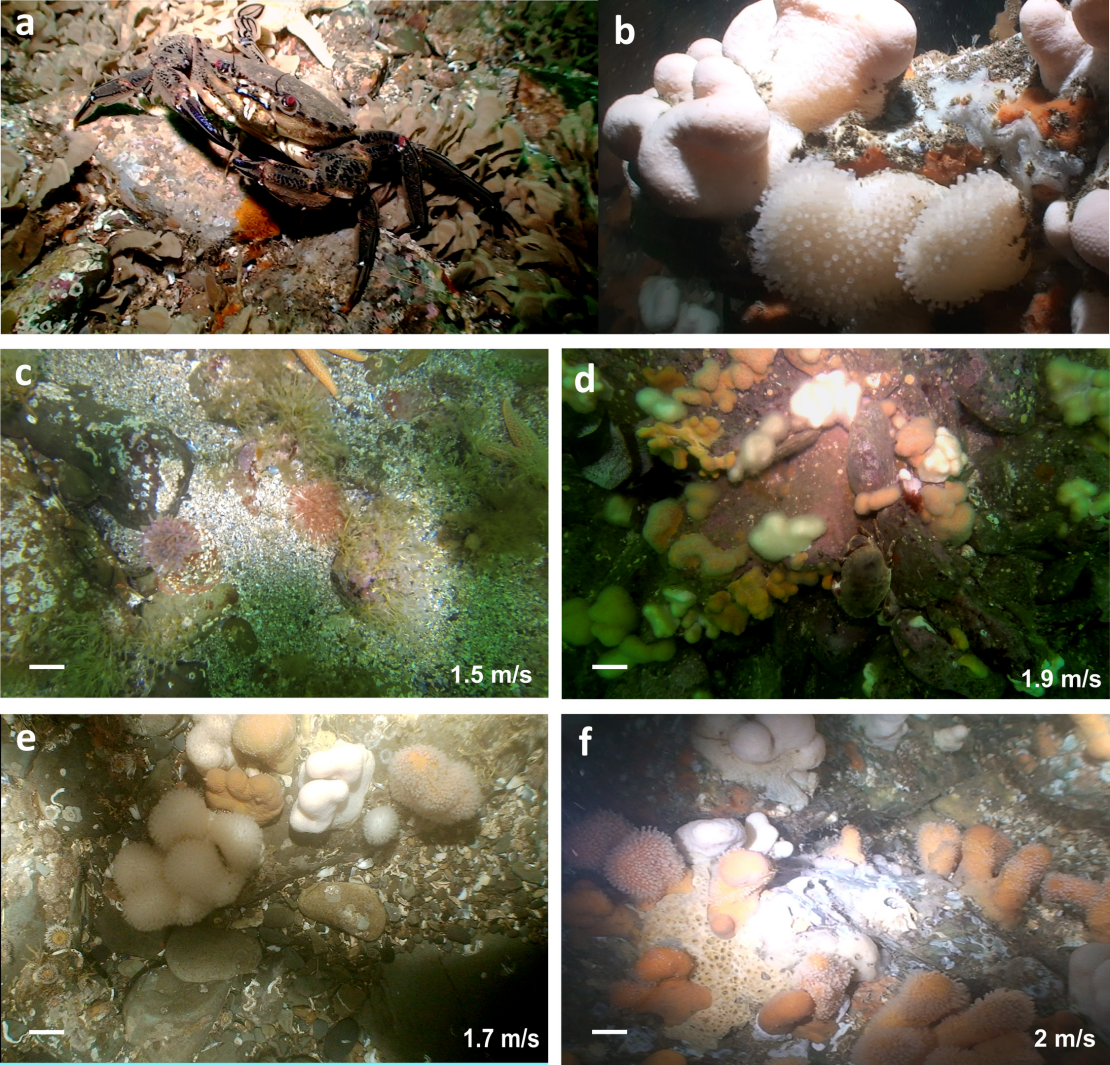
Anemones, soft corals, crustaceans and sponges inhabiting the seafloor of the Strangford Narrows, Strangford Lough. Close up photos of the velvet swimming crab *Necora puber* (a) and dead man’s fingers *Alcyonium digitatum* (b); benthic quadrat images derived from the video footage that were used for the analysis from the sites with flow rates 1.5, 1.7, 1.9 and 2 m/s (c-f). Scale bars represent approximately 0.05 m.

A diverse faunal and limited floral assemblage was observed throughout the Strangford Narrows with a total of 44 taxa recorded (Table 3; Fig 2). The main groups represented were cnidarians, molluscs and bryozoans, with crustaceans, sponges and echinoderms (Table 3). The faunal community of the study area was characterised by the presence of the sponge *Halichondria panicea* and other species of the Phylum Porifera, the polychaete *Spirobranchus* sp. and various members of the Phyla Bryozoa and Cnidaria. Other commonly occurring individual epifauna included the cnidarians *Sagartia* spp., *Alcyonium digitatum*, and *Obelia* spp., as well as representatives of the genus *Balanus* (Fig 2). Several mobile species were also present at > 85% of the sites including the velvet swimming crab *Necora puber* and sea urchin *Echinus esculentus* (Fig 2A & 2D). A range of substratum compositions which matched JNCC recognised biotopes, was recorded throughout the Narrows from cobbles, through small boulder to large boulder all on a bedrock base layer.

**Table 3:** Species list and abundances using the JNCC’s (MNCR) SACFOR abundance scales for each of the 25 sites surveyed by drop down video camera between 25 April and 23 May 2013. S = Superabundant, A = Abundant, C = Common, F = Frequent, O = Occasional, R = Rare.

P50 velocities (m/s)	1.5			1.6			1.7			1.8			1.9			2.0			2.1			2.2			2.4
Replicates	1	2	3	1	2	3	1	2	3	1	2	3	1	2	3	1	2	3	1	2	3	1	2	3	1
**Phylum**																									
**Annelida**																									
*Spirobranchus*	R	O	O	R	O	O	O	O	O	O	O	R	O	O	R	O	O	R	O	O	O	O	O	O	O
*Spirorbis (Spirorbis) spirorbis*	-	-	R	-	-	R	R	-	-	R	-	-	-	R	-	-	-	-	-	-	-	R	-	R	-
**Arthropoda**																									
*Cancer pagurus*	-	R	-	-	R	R	R	R	R	R	R	-	O	R	R	-	R	O	O	R	O	R	R	R	R
*Necora puber*	-	R	-	-	R	R	R	R	R	R	R	R	-	-	R	-	R	R	R	R	R	R	-	R	R
Paguroidea	-	-	-	-	-	-	-	R	R	-	-	-	-	-	-	-	-	-	-	-	-	-	-	-	-
Leptostraca	-	R	-	-	-	-	-	-	R	-	R	-	-	-	-	-	-	-	-	-	-	-	-	-	-
*Balanus*	R	R	R	R	R	R	O	R	O	R	R	R	R	R	R	R	O	R	O	R	R	F	R	F	R
**Bryozoa**																									
*Flustra foliacea*	-	-	F	R	-	-	R	R	-	-	-	R	-	O	-	-	-	R	-	R	-	R	-	-	-
*Alcyonidium*	O	R	-	R	O	R	R	-	-	R	R	-	O	-	-	-	O	-	-	-	-	R	O	O	O
*Alcyonidium diaphanum*	R	O	R	O	O	O	R	R	F	R	O	O	O	F	O	R	R	R	O	-	-	O	R	-	O
Bryozoa	O	O	R	O	O	O	O	O	-	R	R	R	O	O	R	O	O	R	O	O	O	O	F	O	O
**Chordata**																									
*Dendrodoa grossularia*	-	R	-	-	-	-	-	-	-	-	-	R	-	-	R	R	-	R	-	-	-	R	-	-	-
**Cnidaria**																									
*Urticina felina*	-	-	R	-	-	-	-	-	-	-	-	-	-	-	-	-	-	-	-	-	-	-	-	-	-
*Capnea sanguinea*	-	-	R	-	-	-	-	-	-	-	-	-	-	-	-	-	-	-	-	-	-	-	-	-	-
*Sagartia*	R	R	R	O	O	R	R	O	R	R	R	R	R	-	R	R	R	R	R	O	-	R	O	R	R
Actiniaria	-	-	R	-	-	R	-	-	R	-	R	-	R	-	-	-	-	-	-	-	-	-	R	R	-
*Alcyonium digitatum*	R	C	R	F	F	R	O	F	C	O	C	O	O	F	R	O	R	R	F	O	R	F	O	O	R
*Obelia*	-	O	R	-	R	F	R	R	C	O	R	O	C	O	C	R	-	O	O	R	-	O	C	F	C
*Halecium halecinum*	-	-	-	-	-	R	-	-	-	-	-	-	-	O	R	-	-	R	-	-	-	-	-	R	-
*Nemertesia*	-	-	-	R	-	R	-	-	F	-	-	-	-	-	-	-	-	O	R	R	F	-	R	R	-
*Nemertesia antennina*	-	-	-	-	-	-	R	-	-	-	-	-	-	-	-	-	-	-	-	-	-	-	-	-	-
*Abietinaria*	-	-	-	-	-	R	-	-	-	-	-	-	O	-	-	-	-	-	-	-	-	-	O	R	R
*Hydrallmania*	-	R	-	-	-	-	-	-	-	R	-	-	-	-	-	-	R	-	-	-	-	R	-	-	-
Hydrozoa	O	O	R	R	O	O	R	R	F	R	O	R	F	O	R	R	O	F	O	F	O	R	F	O	O
**Echinodermata**																									
*Asterias rubens*	-	R	O	-	R	-	-	-	R	-	R	-	-	R	R	-	R	R	-	-	-	-	-	R	R
*Henricia oculata*	-	-	-	R	R	-	-	-	-	-	-	-	-	R	-	-	-	-	-	-	-	-	-	-	-
*Crossaster papposus*	-	-	-	-	R	-	-	-	-	-	-	-	-	-	-	-	-	-	-	-	-	-	-	-	-
*Echinus esculentus*	-	F	-	R	O	R	R	-	O	R	O	R	R	R	R	R	-	R	R	R	-	R	R	R	R
**Mollusca**																									
*Mytilus edulis*	-	-	-	-	-	R	-	-	-	-	-	-	R	-	-	-	-	-	-	-	-	-	-	R	R
*Venus casina*	-	-	R	-	-	-	-	-	-	-	-	-	-	-	-	-	-	-	-	-	-	-	-	-	-
*Turritella communis*	-	-	-	-	-	-	-	-	-	-	-	R	-	-	-	-	-	-	-	-	R	-	-	-	-
*Buccinum undatum*	-	-	-	R	R	R	O	R	R	-	R	R	R	-	R	-	-	R	-	R	-	-	R	R	R
*Nucella lapillus*	F	-	R	-	R	R	R	R	R	-	R	R	R	R	R	-	C	R	R	O	O	-	R	R	R
*Calliostoma zizyphinum*	R	-	R	-	-	-	-	-	-	-	-	-	-	-	R	-	-	R	R	-	R	-	-	-	R
*Gibbula cineraria*	-	-	R	-	-	-	-	-	-	-	-	-	-	-	-	-	-	-	-	-	-	-	-	-	-
**Ochrophyta**																									
*Laminaria hyperborea*	-	-	-	-	-	-	-	-	-	-	-	-	-	R	R	-	-	-	-	-	-	-	-	-	-
**Porifera**																									
*Axinella*	-	-	-	-	-	-	-	-	-	-	-	-	-	-	-	R	-	-	-	-	-	-	-	-	-
*Halichondria (Halichondria) bowerbanki*	-	-	-	-	-	-	R	-	-	-	-	-	-	-	-	R	-	-	R	-	-	-	-	-	-
*Halichondria (Halichondria) panicea*	O	F	-	F	F	O	F	F	C	F	F	F	F	F	F	F	O	C	C	F	R	F	F	O	F
Porifera	R	F	R	R	O	O	O	R	F	R	F	O	F	O	R	O	R	F	-	O	O	R	F	O	O
**Rhodophyta**																									
*Phyllophora crispa*	-	-	R	-	-	-	-	-	-	-	-	R	-	-	-	-	-	R	-	-	-	-	-	-	-
*Palmaria palmata*	-	-	-	-	-	-	-	-	-	-	-	-	-	-	R	-	-	-	-	-	-	-	-	-	-
Encrusting Coralline Algae	-	R	R	-	-	-	R	R	-	R	-	R	-	R	R	R	-	R	-	-	-	R	-	-	-
Rhodophyta	-	-	R	-	-	-	-	-	-	-	-	-	-	-	-	-	R	-	-	-	-	-	-	-	-

Simper analysis (Table 4) demonstrated that all communities sampled corresponded to the EUNIS biotope CR.HCR.FaT (Very tide-swept faunal communities on circalittoral rock). Permanova analysis (Table 5) showed that when sampling site was nested as a random factor within velocity class, there was no significant effect of the P50 value on macrofaunal community structure. Thus within the narrow channel at the entrance to Strangford Lough, the composition of the epifaunal communities did not differ significantly within the median velocity range of 1.5 to 2.4 m/s, as defined by the P50 value.

**Table 4:** Simper analysis of taxa characterising the sessile epifaunal community of Strangford Lough Narrows.

Species	Av.Abund	Av.Sim	Sim/SD	Contrib%	Cum.%
*Halichondria (Halichondria) panicea*	2.3	9.13	1.21	23.18	23.18
*Spirobranchus*	0.96	6.13	1.55	15.56	38.74
Bryozoa	0.99	4.44	1.3	11.28	50.02
*Alcyonium digitatum*	1.57	4.08	0.61	10.36	60.38
Porifera	1.3	3.02	0.59	7.66	68.04
Hydrozoa	1.22	3.01	0.64	7.65	75.69
*Obelia*	1.36	2.83	0.47	7.19	82.88
*Balanus*	0.81	2.04	0.6	5.18	88.06
*Alcyonidium diaphanum*	0.98	1.56	0.37	3.96	92.02
Average Similarity 34.4%					

**Table 5:** Permanova analysis of Bray Curtis similarity matrix derived from epifaunal distributions in Strangford Lough Narrows measured on a SACFOR scale.

						Unique	
Source	df	SS	MS	Pseudo-F	P(perm)	perms	P(MC)
Velocity	8	1.08E+05	13457	0.89696	0.6637	9881	0.6678
Site (Velocity)	16	2.40E+05	15003	13.343	0.0001	9791	0.0001
Res	500	5.62E+05	1124.4				
Total	524	9.10E+05					

## Discussion

Most benthic ecological studies relating to the effects of anthropogenic disturbance have focussed on the consequences of adding energy to the seafloor in the form of dredging, fisheries-associated abrasion and spoil disposal. In contrast the present study took a reverse approach by focussing on the effects of removing energy from the environment. However few of the studies that have been carried out to establish relationships between current strength and biological distributions have been based on estimates of absolute current speeds, as opposed to qualitative estimates [29, 30]. Two exceptions to the qualitative approach are the studies of Ordines et al. [14] and Dutertre et al. [31] who investigated variance in benthic community distribution and substrate type. The effects of varying mean current flow on the distribution of suspension feeding epifauna such as corals [29] and *Mytilus* beds and sponge dominated communities on vertical rock walls [30] have also been assessed, with it being shown that flow velocities significantly influence the distribution of these suspension feeding communities. A clear link is apparent between the spatial patterns observed for marine communities and substrate type over a wide range of ambient velocities. This conclusion is based on the assumption that substrate is controlled by local ambient velocity (e.g. [12, 13, 14, 15]). In the present study, substrate type was relatively homogenous and no changes in benthic communities at the high end of the velocity spectrum over almost 1 m/s velocity range were observed, this most likely reflecting the highly physically disturbed nature of the environment.

High velocities > 1.5 m/s are characteristic of flow rates found in a number of locations around the UK, with several of these locations being actively sought for exploitation by the tidal energy industry. Habitats in these environments have been described as rich in terms of biodiversity and production [11, 32]. However in comparison to low energy flow environments, here defined as < 1 m/s, few studies have been carried out that quantitatively describe the natural variability of the benthic communities in these high energy flow environments [15, 33, 34] and none directly related to absolute velocities. This is perhaps unsurprising given that marine soft-sediment habitats, associated with low velocity environments, are the most common benthic habitats in the marine environment covering approximately 70% of the planet [35]. The logistical difficulties in sampling high flow environments due to the reduced time available for sampling and limitations in sampling methods [36] will also have contributed to the general lack of detailed investigation of these areas. For example, SCUBA sampling is costly and constrained by the tidal velocity and depth, while grab samples cannot be used on hard substrata such as rock and boulders. Despite the limitations in sampling in these physically challenging environments that may also miss, for example, certain localised fauna such as crevice species, reef systems in these environments have been classified qualitatively as rich in terms of biodiversity and secondary production [11] and can be considered ecologically important environments. In addition, the majority of these high energy environments have been subject to minimal anthropogenic influence.

The species observed in the present study were consistent with those typically recorded for high current flow environments from other comparable benthic studies around the UK [11, 33, 34]. The most common species recorded in the Strangford Narrows were the soft coral dead-men’s fingers *Alcyonium digitatum*, the sponge *Halichondria panacea*, and various members from the Bryozoa, Cnidaria and Porifera Phyla most noticeably *Alcyonidium* spp., *Obelia* spp., *Sagartia* spp., and representatives from the spp. *Hydrozoa* and *Bryozoa* spp. (Table 4). The communities correspond to very tide-swept faunal communities in the EUNIS classification [11, 33, 34]. The present investigation provides valuable insight into the diversity that is present in the Strangford Lough Narrows, but which may also be expected in other comparable areas where resource consent is being sought for tidal energy investment.

The lack of change in the benthic epifauna community over a velocity range of almost 1 m/s presents a challenge for assessing possible effects of tidal energy devices on the local benthic communities in the context of high energy, physically disturbed and highly variable locations [16]. However, despite the apparent homogeneity in the distribution of the benthos recorded in the present study, the environment in the Strangford Narrows is by no means homogenous physically. Variation in flow speed is appreciable over distances as small as 50–100 m with flow speeds varying by almost 0.5 m/s as indicated in red on Fig 1. Overall the lack of a significant effect of current speed on the composition of the benthic communities or distribution of specific taxa suggests that the communities are adapted to the high physical disturbance associated with the strong current flows in the Strangford Narrows. Disturbed boulder habitats are characterised by faunal communities that cover a broad spectrum of successional states. Sousa [37, 38] described spatial community structure on boulder tops as a heterogeneous mosaic, with each boulder top as “a patch of habitat which differs in size and age from that of neighbouring boulders”. These communities therefore could be observed as a mosaic of opportunistic species and individuals being continuously and randomly affected by turbulent forces and physical damage from abrasion and contact with sand and larger sediment particles being transported along the seabed.

The results of this study are of major significance to developers and regulators in the tidal energy industry and may provide a model of the effects of energy removal on community type that can be used to determine when a development has had a significant effect on the local communities. Development of hydrodynamic models predicting the flow perturbations associated with the deployment of tidal energy devices will allow the regulators to relate predicted changes in absolute velocities obtained as output from models to the effects on benthic communities. We also wish to emphasise that the benthic data obtained in the present study is of a form that is typically available to environmental managers when assessing proposed developments. The investigation has shown that the composition of benthic communities is stable over an approximate 1 m/s range of velocities in high velocity flow environments and that, hence, the effects of tidal energy devices on benthic communities in high velocity environments at far-field scales is likely to be minimal.

## Conclusion

At the far-field scale (small water body or environmental impact assessment study area), the relationship between macrobenthic community structure and flow dynamics is likely to depend on local topography, tidal currents and other site specific factors. In this study we have demonstrated a method for high energy epifaunal communities with high levels of natural variability that uses robust replicated analysis to determine the local linkage between flow and community structure that applies to the water body in question. We suggest that studies of this type be incorporated into the ecological assessment of future proposed tidal energy developments. In situ work provides real time information of the system and is highly important to inform and make ecological recommendations for regulators and developers. This would enable environmental mangers to assess the likely impacts of energy removal *per se* on the local communities.
